# Impact of prophylaxis with rituximab on EBV-related complications after allogeneic hematopoietic cell transplantation in children

**DOI:** 10.3389/fimmu.2024.1427637

**Published:** 2024-07-11

**Authors:** Agata Marjańska, Monika Pogorzała, Magdalena Dziedzic, Krzysztof Czyżewski, Monika Richert-Przygońska, Robert Dębski, Tomasz Bogiel, Jan Styczyński

**Affiliations:** ^1^ Department of Pediatric Hematology and Oncology, Nicolaus Copernicus University Torun, Collegium Medicum, Bydgoszcz, Poland; ^2^ Department of Microbiology, Collegium Medicum, Nicolaus Copernicus University Torun, Bydgoszcz, Poland

**Keywords:** EBV infection, PTLD, children, hematopoietic cell transplantation, prophylaxis, transplant outcomes

## Abstract

**Background:**

Children undergoing allo-HCT are at high risk of EBV-related complications. The objective of the study was to analyze the impact of prophylactic post-transplant rituximab on EBV infection and EBV-PTLD in children after allo-HCT, to determine the risk factors for the development of EBV infection and EBV-PTLD and to determine their outcomes. Additionally, the impact of EBV-driven complications on transplant outcomes was analyzed.

**Methods:**

Single center retrospective analysis of EBV-related complications in pediatric population undergoing allo-HCT, based on strategy of prophylaxis with rituximab. Overall 276 consecutive children, including 122 on prophylaxis, were analyzed for EBV-driven complications and transplant outcomes.

**Results:**

Prophylaxis with rituximab resulted in significant reduction of EBV infection (from 35.1% to 20.5%; HR=2.7; p<0.0001), and EBV-PTLD (from 13.0% to 3.3%; HR=0.23; p=0.0045). A trend for improved survival was also observed (HR=0.66; p=0.068), while non-relapse mortality was comparable in both cohorts. The peak value of viral load was a risk factor in the development of EBV-PTLD: 10-fold higher peak viral load in comparison to the baseline 10^4^ copies/mL, caused a 3-fold (HR=3.36; p<0.001) increase in the risk of EBV-PTLD. Rituximab treatment was effective as a preemptive therapy in 91.1%, and in 70.9% in EBV-PTLD. Patients who developed PTLD had dismal 5-year overall survival (29% vs 60%; p<0.001), and an increased risk of relapse (72% vs 35%; p=0.024).

**Conclusions:**

Rituximab for prophylaxis of EBV infection and EBV-PTLD was highly effective in pediatric population. Treatment of EBV-PTLD was successful in 70%, however the occurrence of EBV-PTLD was associated with an increased risk of relapse of primary malignant disease.

## Introduction

Epstein-Barr virus (EBV, HHV-4) is a DNA virus belonging to the *Herpesviridae* family, with a very high prevalence. As a latent virus, the virus is localized in B lymphocytes and remains long-life in the body without causing significant clinical symptoms. Primary infection occurs most often in early childhood. Most adults worldwide (85%) are seropositive (1–3).

In healthy individuals, there is a balance between EBV-infected B lymphocytes and T lymphocytes that act as immune surveillance. Immunosuppressed patients are at high risk of viral reactivation and the development of EBV-related disease. The clinical picture of primary infection or reactivation can be manifested by various diseases (4–6).

Post-transplant EBV reactivation manifests as EBV-DNA-emia which may be followed by fever and lymphadenopathy. The incidence of EBV reactivation after hematopoietic cell transplantation (HCT) ranges from 0.1% to 63% (7). The most severe form of EBV reactivation is post-transplant lymphoproliferative syndrome (PTLD), which 20 years ago was associated with a mortality rate of 86% (8), and currently decreased to 30-50%, although the outcome seems to be better in children than adults (9–14).

Management of EBV-PTLD in hematopoietic cell transplantation setting, based on the ECIL-6 guidelines for monitoring and prevention, allows for early detection of EBV reactivation and the implementation of appropriate treatment. With these recommendations, rituximab was shown to have 90% efficacy in preemptive therapy and 65% efficacy in EBV-PTLD therapy (7). A reduction of immunosuppression in combination with rituximab increases the effectiveness of treatment up to 78% (9). Similar outcomes were shown with the use of EBV-specific cytotoxic T lymphocytes (EBV-CTLs), with 90% of cure rate in preemptive therapy and 75% in EBV-PTLD therapy (4). However, the mortality rate due to PTLD is approximately 20-25%.

Prevention of EBV reactivation includes the use of drugs in asymptomatic, EBV-seropositive patients to prevent the occurrence of EBV-DNA-emia. Administration of rituximab (anti-CD20 monoclonal antibody) before or shortly after HCT depletes B cells and thus may reduce the risk of EBV-DNA-emia and PTLD. In 2012, Dominietto et al. show a significant reduction in the incidence of EBV-DNA-emia after using rituximab on day +5 after HCT in adults (15). In two other studies on the prophylactic role of rituximab in adults, Van Besien et al. (16) and Patel et al. (17) used one pretransplant dose of rituximab. Van Besien et al. (16) administered a prophylactic dose of rituximab 375 mg/m2 pre-transplant in haplo-cord transplantation which combined a mismatched UCB graft with third-party cells. They used thymoglobulin in conditioning. Patients who did not receive rituximab, had the cumulative incidence of post-transplant EBV reactivation and of EBV PTLD was 13% and 8%, respectively, while those who received pre-transplant rituximab, the incidences were 2% (p=0.0017) and 0% (p=0.04), respectively. As there was no difference in time to hematopoietic recovery, in the incidence of CMV reactivation, of invasive blood stream infections or of proven or probable invasive fungal infections, pre-transplant administration of rituximab was an effective and non-toxic intervention that drastically reduced EBV reactivation and PTLD in high-risk patients. In the study of Patel et al. (17) the primary endpoint was incidence of EBV reactivation at day 180 among adults who had allo-HCT with *in vivo* T-cell depletion with alemtuzumab receiving pre-HCT rituximab versus those not receiving rituximab. EBV reactivation at day 180 occurred in 23 (53%) patients without prior rituximab exposure versus 0 patients with pre-HSCT rituximab exposure (p<0.0001), so the administration of pre-HCT rituximab before allo-HCT in adult patients receiving T-cell depletion with alemtuzumab was associated with a significant decrease in the risk for EBV reactivation and EBV-PTLD, without increasing aGVHD or infection rates.

Children undergoing allo-HCT are at high risk of EBV-related complications. The objective of the study was to analyze the efficacy of strategy of prevention of EBV infections, based on prophylaxis with rituximab in children after allo-HCT, to determine the risk factors for the development of EBV infection and EBV-PTLD and to determine their outcomes. Additionally, the impact of EBV-driven complications on transplant outcomes was analyzed.

## Patients and methods

### Design of the study

Single center retrospective analysis of EBV infection and EBV-PTLD in pediatric population undergoing allogeneic HCT over a period of 13 years, with the implementation of strategy of prophylaxis with rituximab in anti-EBV management.

### Strategy of EBV management

Management of prevention of EBV infection in children after allo-HCT was based on screening for EBV-DNA by PCR and pre-emptive therapy for EBV-DNA-emia. Over the study period, a strategy of prevention was implemented based on prophylaxis with rituximab. Between 2008-2015 (Group A), EBV-DNA viral load was monitored in different intervals and pre-emptive treatment was applied, when the viral load was ≥10^4^ copies/mL. Between 2015-2020 (Group B), the monitoring of EBV-DNA viral load was performed weekly and it was preceded by administration of rituximab used for prophylaxis at a dose of 150 mg/m^2^ (max. 200 mg), on day +5 after allo-HCT. The dose of rituximab was based on study of Dominietto et al, adjusted to pediatric setting (15). In case of viral load ≥10^4^ copies/mL, pre-emptive therapy was introduced. All patients were included in the prophylaxis, as EBV-PTLD in HCT setting is almost exclusively of recipient origin, and children are at high risk of primary EBV infection.

### EBV diagnostics

Before hematopoietic cell transplantation, recipients and donors were screened for EBV serological markers (anti-EBNA; EBV-IgG, EBV-IgM). After transplantation, the diagnosis of infection was carried out using the quantitative real-time polymerase chain reaction (real-time qPCR; CFX96 Touch Real-Time PCR Detection System, Bio-Rad; or Roche’s Cobas z480) method from plasma samples, and in the case of suspected central nervous system infection, also from the cerebrospinal fluid. For the purpose of this analysis, the quantitative results of EBV-DNA-emia were rounded down to the full power and the cut-off point for detection of EBV-DNA-emia was 3x10^2^ copies/mL. In the case of suspected EBV-PTLD, depending on the patient’s clinical condition, the diagnostics was extended to biopsy of the tumor and/or PET-CT/CT/MRI imaging.

**Definitions:**

- EBV infection was diagnosed in case of virus isolation or detection of viral nucleic acid (NAT, nucleic acid test) or antigens (proteins) in any body fluid or tissue specimen.
- Primary EBV infection was defined with first detected of EBV in an individual who had no evidence of EBV exposure.
- EBV replication indicates evidence of viral multiplication and presence of EBV-DNA.
- EBV-DNA-emia (historically referred also as reactivation or latent infection) – detection of any EBV-DNA in the plasma (with or without fever, but with no sign of EBV end-organ disease).
- Clinically significant EBV-DNA-emia (csEBV-DNA-emia; referred also as csEBV infection, csEBVi) – viremia, which required implementation of pre-emptive therapy. We assumed the value of EBV-DNA-emia ≥10^4^ copies/mL as the threshold value for implementation of pre-emptive therapy.
- EBV-associated post-transplant lymphoproliferative disorder (EBV-PTLD) was referred as symptomatic disease, with EBV-associated post-transplant manifestations.
- EBV-PTLD is a life-threatening complication after allogeneic transplantation of hematopoietic cells. PTLD is a heterogeneous group of lymphoproliferative diseases that occur in the course of transplantation and result from uncontrolled neoplastic proliferation of lymphoid or plasma cells as a result of iatrogenic suppression of T lymphocytes. From the clinical point of view, PTLD can be distinguished at a proven or probable level of diagnosis (7, 18).
- Proven EBV-PTLD was diagnosed in case of presence of symptoms and/or signs from the affected organ together with detection of EBV nucleic acids or EBV-encoded proteins in a tissue specimen obtained from an organ by biopsy or other invasive procedures with a test with appropriate sensitivity and specificity together with symptoms and/or signs from the affected organ. Probable EBV disease was diagnosed as significant lymphadenopathy, hepatosplenomegaly, or other end-organ manifestations (without tissue biopsy, but in the absence of other documented cause) together with significant EBV-DNA-emia (7).
- Morphological types of PTLD. According to the 2016 WHO classification, six morphological types of PTLD were distinguished: plasmacytic hyperplasia, infectious mononucleosis, florid follicular hyperplasia, polymorphic, monomorphic (B-cell and T-/NK-cell types), and classical Hodgkin lymphoma (19). A significant change in classification was made in 2022. PTLDs are not any longer listed in the WHO classification of lymphoid malignancies, neither in the International Consensus Classification of Mature Lymphoid Neoplasms. Currently, those tumors are more broadly defined as immunodeficiency-associated lymphoproliferative disorders (20) or lymphoid proliferations and lymphomas associated with immune deficiency and dysregulation (21).
- First diagnosis was assumed as the primary disease, being an indication for allo-HCT.

### Prophylaxis, pre-emptive and targeted treatment of EBV complications

EBV-related infections, prevention and treatment strategies, and response to therapy were classified based on the definitions and guidelines of the European Conference on Infections in Leukemia (ECIL) (7, 18). Patients with csEBV-DNA-emia were eligible for pre-emptive treatment with rituximab administered weekly until two negative results of EBV-DNA-emia, and reduction of immunosuppression (RI), if possible. Therapy included an intravenous infusion of rituximab (anti-CD20 monoclonal antibody) at a dose of 375 mg/m^2^. The end of follow-up was June 2020.

Treatment of EBV-PTLD included weekly rituximab at a dose of 375 mg/m^2^, and a reduction of immunosuppression (RIS), if possible. For second line of therapy of EBV-PTLD, chemotherapy R-CHOP, DLI (donor lymphocyte infusion) or EBV-CTL (EBV-specific cytotoxic T-lymphocytes) were considered, if available. A reduction of immunosuppression was defined as a sustained decrease of at least 20% of the daily dose of immunosuppressive drugs with the exception of low-dose corticosteroid therapy (7). Initial response to rituximab was defined as response to rituximab therapy identified by a decrease in EBV-DNA-emia of at least 1 log10 after first two weeks of treatment.

### Anti-infective prophylaxis

Antiviral, antibacterial and antifungal prophylaxis were administered to all patients after hematopoietic cell transplantation in accordance with accepted standards (22). The occurrence of invasive fungal disease (IFD) in the pre- and post-transplant period was analyzed. IFD was diagnosed based on current recommendations (23, 24).

### Availability of data and materials

The dataset supporting the conclusions of this article is available from the corresponding author, upon reasonable request.

### Institutional review board statement

The study was conducted in accordance with the Declaration of Helsinki, and approved by the Institutional Review Board (approval numbers: KB 499/2014; KB 696/2017; KB 263/2022). All patients or their parents provided their consent for reporting data related to their treatment.

### Statistical analysis

Non-categorical variables were compared with the Mann-Whitney U test, and categorical variables with the χ^2^ test (Yates correction or Fisher test was applied when necessary). Odds ratios (OR) with 95% confidence intervals (95%CI) were calculated; OR>1 defined increased risk. The cumulative incidences of csEBV-DNA-emia and EBV-PTLD were estimated to be an event of interest, while death without csEBV-DNA-emia or EBV-PTLD was a competing event. The cumulative incidence was computed in a competing risks setting, and the Gray test was used to compare groups. Overall survival (OS) and relapse incidence (RI) were determined by the Kaplan-Meier method and compared by the log-rank test. Overall survival was determined as time from the day of HCT to the occurrence of death or the end of follow-up. Relapse was considered as recurrence of the primary disease after transplantation. Risk factor analyses were performed separately for csEBV-DNA-emia, EBV-PTLD and overall survival. Univariate analyses for risk factors were performed using the Cox method. Factors with p-value in the univariate analysis <0.1 were included in the Cox model in respective multivariate analysis. The hazard ratio (HR) with 95%CI and p-values ​​were calculated for each factor. For acute and chronic GvHD, infections after HCT, and initial response to rituximab, Cox time-dependent analysis was applied. On the basis of the models, a “risk factor index” was created in the form of cumulative incidences depending on the number of risk factors. We analyzed following risk factors: prophylaxis with rituximab (yes vs no), sex (male vs female), age (≥10 vs <10 yrs), diagnosis of acute lymphoblastic leukemia (ALL) (yes vs no), diagnosis (malignant vs non-malignant disease), status of remission (complete remission CR1 vs >CR1), number of HCT (first vs subsequent i.e. >1), type of donor (MUD, matched unrelated donor; MMUD, mismatched unrelated donor; MFD, matched family donor; HAPLO, haploidentical donor), cell source (PB, peripheral blood; BM, bone marrow; CB, cord blood), pretransplant EBV IgG serostatus of donor (D) and recipient (R), D/R pretransplant CMV (cytomegalovirus) IgG serostatus, ABO blood group compatibility (yes vs no), Rh blood group compatibility (yes vs no), conditioning (MAC, myeloablative conditioning; RIC reduced intensity conditioning; TBI, total body irradiation; chemotherapy), T-cell depletion *in vivo* (yes vs no), type of T depletion (ATG, anti-thymocyte globulin vs alemtuzumab), CMV infection (yes vs no), BKV (polyomavirus BK) infection (yes vs no), invasive fungal infection (IFI) before/after HCT (yes vs no), acute graft versus host disease (aGvHD) (yes vs no), chronic GVHD (cGvHD) (176 pts evaluable) (yes vs no), time from HCT to EBV infection (<100 days vs ≥100 days), maximal value of EBV-DNA-emia (copies/mL) (<10^5^ vs ≥10^5^), good response after 2 doses of rituximab in pre-emptive therapy (yes vs no), EBV-DNA-emia (yes vs no), and EBV-PTLD (yes vs no). A significance level of p<0.05 was assumed in the study.

## Results

### Demographics

A total of 276 consecutive patients under the age of 18 on the day of the first diagnosis, after allo-HCT between 2007-2020 were included in the study. The median age at diagnosis of the primary disease was 7.0 years (min-max, 0.1-17.9). The median age at transplant was 9.9 years (min-max 0.3-22.0). Detailed patient characteristics are presented in **Table 1**. Patients who received prophylaxis with rituximab (group B) were younger (median age 8.0 vs 10.8 years), had more MUD transplants, and more often received ATG.

**Table 1:** Patient characteristics.

Parameter		Total N=276	Group A N=154	Group B N=122	p-value
Sex	Female	105 (38.0%)	56 (36.4%)	49 (40.2%)	0.894
	Male	171 (62.0%)	98 (63.6%)	83 (59.9%)	
Age at transplant	<10 years	139 (50.4%)	68 (44.2%)	71 (58.2%)	0.028
	≥10 years	137 (49.6%)	86 (55.8%)	51 (41.8%)	
	Median, min-max	9.9 (0.3–22.0)	10.8 (0.3-22)	8.0 (0.7-19.8)	0.007
Diagnosis	Malignant: acute leukemias and MDS (74.2%), other malignant (6.0%)	225	125 (81.1%)	100 (82.0%)	0.865
	Non-malignant: SAA/BMF (12.3%), IEI (7.5%)	51	29 (18.9%)	22 (18.0%)	
Remission status at transplant*	CR1	157 (56.9%)	85 (55.2%)	72 (59.0%)	0.524
	>CR1	119 (43.1%)	69 (44.8%)	50 (41.0%)	
Type of donor	MUD	195 (70.6%)	98 (63.6%)	97 (79.6%)	0.016
	MFD	67 (24.3%)	45 (29.2%)	22 (18.0%)	
	MMUD	9 (3.3%)	8 (5.2%)	1 (0.8%)	
	HAPLO	5 (1.8%)	3 (2.0%)	2 (1.6%)	
Cell source	Peripheral blood	178 (64.5%)	93 (60.3%)	85 (69.7%)	0.163
	Bone marrow	95 (34.4%)	58 (37.7%)	37 (30.3%)	
	Cord blood	3 (1.1%)	3 (2.0%)	0	
Conditioning	MAC	163 (59.1%)	95 (61.7%)	68 (55.7%)	0.318
	RIC	113 (40.9%)	59 (38.3%)	54 (44.3%)	
	TBI	46 (16.7%)	28 (18.2%)	16 (13.1%)	0.253
T-depletion *in vivo*	ATG	212 (76.8%)	105 (68.2%)	107 (87.7%)	<0.001
	Alemtuzumab	11 (4.0%)	11 (7.1%)	0	0.004
GvHD prophylaxis	CsA-based	267 (96.8%)	148 (96.1%)	120 (98.4%)	0.267
	PTCy	4 (1.4%)	0	4 (3.3%)	
EBV serostatus	R–/D–	3 (1.1%)	1 (0.6%)	2 (1.6%)	0.235
	R–/D+	14 (5.1%)	8 (5.2%)	6 (4.9%)	
	R+/D–	20 (7.2%)	10 (6.5%)	10 (8.2%)	
	R+/D+	181 (65.6%)	77 (50.0%)	104 (85.2%)	
	ND	58 (21.0%)	58 (37.6%)	0 (0%)	
Donor EBV IgG	positive	212 (76.8%)	102 (66.2%)	110 (90.2%)	0.979
	negative	23 (8.3%)	11 (7.1%)	12 (9.8%)	
	ND	41 (14.9%)	41 (26.7%)	0	
Recipient EBV IgG	positive	208 (75.4%)	94 (61.1%)	114 (93.4%)	0.215
	negative	21 (7.6%)	13 (8.4%)	8 (6.6%)	
	ND	47 (17%)	47 (30.5%)	0	
aGvHD	Yes	96 (34.8%)	59 (38.3%)	37 (30.3%)	0.166
cGvHD (176 pts evaluable)	Yes	40 (14.5%)	30 (19.5%)	10 (8.2%)	0.013

* non-malignant diseases were classified as CR1; ND, no data; SAA, severe aplastic anemia; BMF, bone marrow failure; IEI, inborn errors of immunity; CR, complete remission; MFD, matched family donor; MUD, matched unrelated donor; MMUD, mismatched unrelated donor; HAPLO, haploidentical donor; MAC, myeloablative conditioning; RIC, reduced intensity of conditioning; TBI, total body irradiation; ATG, anti0thymocyte globulin; CsA, cyclosporin A; PTCy, post-transplant cyclophosphamide; GvHD, graft-versus-host disease; aGvHD, acute GvHD; cGvHD, chronic GvHD; R, recipient; D, donor; ND, not done.

### Clinically significant EBV-DNA-emia (csEBV-DNA-emia)

**Figure 1:**
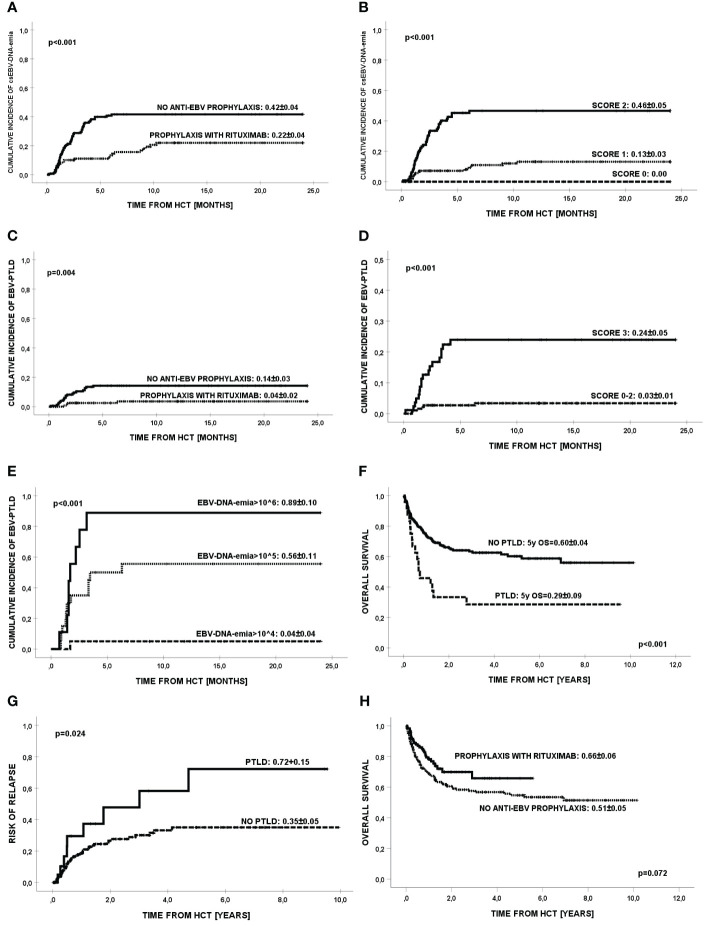
EBV-related complications: **(A)** Cumulative rates of first csEBV-DNA-emia by treatment regimen; **(B)** Prognostic model for csEBV-DNA-emia depending on the number of unfavorable prognostic factors (risk factors: lack of rituximab for prophylaxis, T cell depletion *in vivo*; categories: 0, 1, 2); **(C)** Cumulative incidence of EBV-PTLD; **(D)** Prognostic model for the development of EBV-PTLD depending on the number of unfavorable risk factors (risk factors: no use of rituximab in prophylaxis, MUD/MMUD donor, hematopoietic cell transplantation from peripheral blood); **(E)** Cumulative incidence of EBV-PTLD according to peak value of EBV-DNA-emia [copies/mL]; **(F)** Overall survival in patients with/without EBV-PTLD; **(G)** Risk of relapse of primary disease by EBV-PTLD (malignant diseases only); **(H)** Overall survival in patients with/without anti-EBV prophylaxis with rituximab.

The total prevalence of csEBV-DNA-emia was 28.6%, including 37.0% in group A, and 18% in group B (HR=2.67; 95%CI=1.52-4.70; p<0.001) (**Table 2**). The cumulative incidence of first EBV reactivation is shown in **Figure 1A**. The median time to diagnosis of csEBV-DNA-emia after allo-HCT was 70 days (IQR, 41-122). Over 67% of patients were diagnosed with csEBV-DNA-emia within 100 days of HCT. Overall, 35.4% of patients with EBV reactivation had a peak viral load ≥10^5^ copies/mL and the remaining 64.6% patients had a peak viral load <10^5^ copies/mL. Primary csEBV infections occurred in 7/14 EBV-seronegative recipients from EBV-seropositive donors.

**Table 2:** Prevalence and duration of csEBV-DNA-emia.

Parameter	Total	Group A	Group B	p-value
Number of patients	276	154	122	
Median number of PCR tests per patient (IQR)	12 (9-18)	8 (6-18)	16 (12-19)	<0.001
csEBVi prevalence	79 (28.6%)	57 (37.0%)	22 (18.0%)	<0.001
Median time (IQR)	70 (41-132)	66 (30-116)	89 (35-132)	0.018
Patients with time to viremia <100 days	53 (67.1%)	41 (71.9%)	12 (54.5%)	0.140
Value of peak EBV-DNA-emia ≥10^5^ copies/mL	28 (35.4%)	22 (38.6%)	6 (27.3%)	0.495
Value of peak EBV-DNA-emia <10^5^ copies/mL	51 (64.6%)	35 (61.4%)	16 (72.7%)	

IQR, interquartile range.

### Risk factor analysis for csEBV-DNA-emia

In univariate analysis, factors significantly associated with the development of csEBV-DNA-emia were: source of hematopoietic cells (PB vs BM; HR=2.03; 95%CI=1.12-3.68; p=0.0174), *in vivo* T-cell depletion with ATG (HR=13.3; 95%CI=3.14-54; p<0.0001), and chronic GVHD (HR=2.03; 95%CI=1.03-3.99; p=0.0366), while use of rituximab in prophylaxis (HR=0.48; 95%CI=0.28-0.83; p=0.0078), and MFD donor (MFD vs other; HR=0.21; 95%CI=0.09-0.49; p=0.0005) decreased the risk (**Supplementary Table S1**).

In multivariate analysis, following factors significantly contributed to the risk of csEBV-DNA-emia: the use of rituximab for prophylaxis (HR=0.35; 95%CI=0.21-0.56; p<0.0001) and T-cell depletion *in vivo* with ATG (HR=16.7; 95%CI=4.0-50.0; p<0.0001) (**Table 3**). The use of rituximab for prophylaxis and the lack of T-cell depletion *in vivo* reduced the risk of EBV reactivation. Based on the above risk factors, a prognostic model was created for csEBV-DNA-emia patients (**Figure 1B**). Unfavorable prognostic factors included lack of rituximab prophylaxis and T cell depletion *in vivo*. In patients with no listed risk factors, csEBV-DNA-emia did not occur.

**Table 3:** Multivariate analysis of risk factors for csEBV-DNA-emia.

Parameter		N	csEBV-DNA-emia		p-value	HR	95% CI
			Yes	No			
Patients		276	79 (28.6%)	197 (71.4%)			
Prophylaxis with rituximab	Yes	122	25 (20.5%)	97 (79.5%)	<0.0001	0.35	0.21-0.56
	No	154	54 (35.1%)	100 (64.9%)		1.00	
T-depletion *in vivo*	No	53	2 (3.7%)	51 (96.3%)	<0.0001	16.7	4.0-50.0
	ATG	212	72 (34.0%)	140 (66.0%)		1.00	
	Alemtuzumab	11	5 (45.5%)	6 (54.5%)	ns		

N, number of patients; p, p-value for the χ2 test; HR, hazard ratio; 95%CI, 95% confidence interval for HR (confidence interval). ns; non-significant.

### EBV-PTLD

EBV-PTLD developed in 24/276 (8.7%) patients, including 13.0% (20/154) in group A, and 4/122 (3.3%) in group B. Cumulative incidence of EBV-PTLD is shown on **Figure 1C**. The median time to diagnosis of EBV-PTLD for the entire group was 43 days (min-max, 5-189); 19/24 (79.2%) patients developed EBV-PTLD before day +100 after HCT. Overall, 20/24 (83.3%) patients developed EBV-PTLD with a baseline EBV-DNA-emia ≥10^5^ copies/mL. All patients (n=24) had lymph node involvement, and 9/24 (37.5%) had extranodal involvement. Central nervous system disease was diagnosed in 8/24 patients (33.3%). Multifocal disease was diagnosed in 12/24 patients (50%). EBV-PTLD was biopsy confirmed in 5 patients: 3 patients had the monomorphic form (diffuse large B-cell lymphoma), one had the polymorphic form (n=1), and one had the classic form (n=1). In 19/24 (79.2%) patients, EBV-PTLD was diagnosed at the probable level.

### Risk factor analysis for the development of EBV-PTLD

In univariate analysis, factors significantly associated with the development of EBV-PTLD included: rituximab in prophylaxis (HR=0.23; 95%CI-0.08-0.69; p=0.0045), donor type (MFD vs others; HR=0.13; 95%CI=0.02-0.95; p=0.0463), source of hematopoietic cells (PB vs BM; HR=6.5; 95%CI=1.51-28.5; p=0.0144), T-cell depletion *in vivo* with ATG (HR undetermined; p=0.0046), time from HCT to EBV reactivation (<100 days; HR=48; 95%CI=13.5-170; p=0.0088), EBV-DNA peak viral load <10^5^ copies/mL (HR=0.05; 95%CI=0.02-0.17; p<0.0001), and response to rituximab treatment after 2 doses of rituximab in preemptive therapy had preventive value (HR undetermined; p<0.0001) (**Supplementary Table S1**).

In multivariate analysis, the use of rituximab in prophylaxis 5-fold reduced the risk of developing of EBV-PTLD (p=0.0025). Other factors significantly contributing to prevent PTLD were: MFD donor type (HR=0.12; p=0.0120) and the use of BM as a source of hematopoietic cells (HR=0.21; p=0.0410) (**Table 4**). Based on these three risk factors, a prognostic model for the development of EBV-PTLD was created. Unfavorable prognostic factors included: no prophylaxis with rituximab, MUD/MMUD donor and peripheral blood as a source of cells. In patients with 0-2 risk factors, EBV-PTLD developed in 3%, while when 3 of these factors were present, 24% of patients developed EBV-PTLD (**Figure 1D**). The peak viral load was shown to be another significant factor in the development of EBV-PTLD (p<0.001). A 10-fold higher viral load in comparison to the baseline 10^4^ copies/mL, increased the risk of developing EBV-PTLD more than 3-fold (HR=3.36; 95%CI=2.25-5.03) (**Figure 1E**).

**Table 4:** Multivariate analysis of risk factors for EBV-PTLD.

Parameter		N	EBV-PTLD		p-value	HR	95% CI
			Yes	No			
Patients		276	24 (8.7%)	252 (91.3%)			
Prophylaxis with rituximab	Yes	122	4 (3.3%)	118 (96.7%)	0.0025	0.19	0.07-0.56
	No	154	20 (13.0%)	134 (87.0%)		1.00	
Type of donor	MFD	67	1 (1.5%)	66 (98.5%)	0.0120	0.12	0.02-0.90
	MUD/MMUD	204	23 (11.3%)	181 (88.7%)		1.00	
Cell source	PB	178	22 (12.4%)	156 (87.6%)	0.0410	1.00	0.05-0.94

N, number of patients; HR, hazard ratio; 95%CI, 95% confidence interval; MFD, matched family donor; MUD, matched unrelated donor; MMUD, mismatched unrelated donor; PB, peripheral blood; BM, bone marrow.

### Efficacy of prophylaxis with rituximab

Overall, 44.2% (122/276) of patients received rituximab for prophylaxis. Compared with those without prophylaxis, lower incidences of csEBV-DNA-emia (p=0.0005) and EBV-PTLD (p=0.0045) were observed in patients who received rituximab prophylaxis. The use of rituximab on day +5 after HCT resulted in preventing csEBV-DNA-emia in 79.5% patients (97 out of 122 patients on prophylaxis, had no csEBV-DNA-emia), and in preventing EBV-PTLD in 96.7% patients (118 out of 122 had no EBV-PTLD) (**Table 5**).

**Table 5:** Effect of rituximab prophylaxis on the prevalence of EBV infections and other complications.

Parameter		N	Prophylaxis with rituximab		p-value
			Yes (n=122)	No (n=154)	
csEBV-DNA-emia	Yes	79	22 (18.0%)	57 (37.0%)	0.0005
	No	197	100 (82.0%)	97 (63.0%)	
EBV-PTLD	Yes	24	4 (3.3%)	20 (13.0%)	0.0045
	No	252	118 (96.7%)	134 (87.0%)	
Day of neutrophil recovery (median; IQR)			16 (13-22)	16 (13-23)	0.525
Day of platelet recovery (median; IQR)			19 (15-26)	18 (14-27)	0.317
Rejection/graft failure			2/122	3/154	0.841
CMV infection (N) up to day +180			54 (44.3%)	56 (35.4%)	0.183
Invasive fungal disease (N) up to day +180			34 (27.9%)	41 (26.6%)	0.817
On hematological recovery:					
IgG g/L (median; IQR)			4.7 (2.5-13.1)	4.8 (2.3-14.6)	0.536
IgA g/L (median; IQR)			0.35 (0.12-3.22)	0.37 (0.11-1.82)	0.413
IgM g/L (median; IQR)			0.37 (0.10-3.04)	0.33 (0.12-2.17)	0.680
On day +100:					
IgG g/L (median; IQR)			4.5 (3.7-10.8)	4.8 (3.1-12.5)	0.704
IgA g/L (median; IQR)			0.33 (0.06-3.36)	0.37 (0.09-2.65)	0.542
IgM g/L (median; IQR)			0.28 (0.14-3.12)	0.31 (0.07-2.93)	0.529
On day +180:					
IgG g/L (median; IQR)			4.1 (2.3-11.3)	4.2 (2.8-14.4)	0.603
IgA g/L (median; IQR)			0.29 (0.12-2.32)	0.35 (0.09-1.56)	0.819
IgM g/L (median; IQR)			0.30 (0.12-2.55)	0.33 (0.08-1.68)	0.758
CD19+ count >0.2×10^9^/L on day +180 (among available patients)			31/58 (53.4%)	23/65 (35.4%)	0.043

N, number of patients.

### Treatment of csEBV-DNA-emia and EBV-PTLD

#### Preemptive treatment

Overall, 79 patients with csEBV-DNA-emia received preemptive treatment with rituximab: 57/154 (37%) in group A and 22/122 (18%) in group B (**Table 6**). The median number of rituximab doses was 2 (IQR: 1-4), and 52/79 (65.8%) patients achieved a decrease of EBV viremia by at least 1 log10 (i.e. 10-fold) after 2 weeks of treatment.

**Table 6:** Treatment of csEBV-DNA-emia and EBV-PTLD by treatment regimen.

Patients with csEBV-DNA-emia		Group A	Group B	Total
Number of patients		154	122	276
Treatment	Preemptive treatment with rituximab	57 (37.0%)	22 (18.0%)	79 (28.6%)
	Number of rituximab doses: median, IQR	2 (1-4)	4 (1-4)	2 (1-4)
Reduction of immunosuppression	Yes	21 (36.8%)	6 (27.3%)	27 (34.2%)
	No	36 (63.2%)	16 (72.7%)	52 (65.8%)
Response after 2 doses of rituximab	↓ viral load	36 (63.2%)	16 (72.7%)	52 (65.8%)
	Stable or ↑ viral load	21 (36.8%)	6 (27.3%)	27 (34.2%)
EBV-PTLD	Yes	20 (35.1%)	4 (18.2%)	24 (30.4%)
	No	37 (64.9%)	18 (81.8%)	55 (69.6%)
	Treatment with rituximab	20	4	24
	Number of doses of rituximab: median, IQR	4 (4-4)	4 (4-4)	4 (4-4)
	Reduction of immunosuppression	20	4	24
	Intrathecal rituximab	8 (14.0%)	0 (0.0%)	8 (10.1%)
	Chemotherapy	1 (5%)	0 (0.0%)	1 (4%)

↓, decrease; ↑, increase.

#### EBV-PTLD

EBV-PTLD was diagnosed in 30% of patients with csEBV-DNA-emia (24/79). EBV-PTLD was significantly more frequent when pre-emptive treatment was started in patients with a viral load ≥10^5^ than in those with a viral load <10^5^ copies/mL (**Table 7**). Preemptive therapy was successful in 72/79 (91.1%); as 7 patients died due to EBV-PTLD. Intravenous rituximab was used to treat EBV-PTLD; in 8/24 patients EBV-DNA was present in CNS fluid, so rituximab was also applied intrathecally. Immunosuppressive treatment was reduced in all patients with EBV-PTLD. Therapy of EBV-PTLD led to resolution of PTLD in 17/24 (70.9%) patients; 6 patients died during rituximab therapy, and one patient was eligible for second-line treatment with R-CHOP chemotherapy, but died from progression of PTLD (**Table 8**). EBV-CTL and DLI and were not used for therapy of EBV-PTLD in this cohort.

**Table 7:** Evaluation of EBV complications and EBV-attributed deaths according to viral load.

Parameter	N	Peak value of EBV-DNA-emia (copies/mL)		p-value
		<10^5^	≥10^5^	
csEBV-DNA-emia	79	51 (64.6%)	28 (35.4%)	
EBV-PTLD	24	3/51 (5.9%)	21/28 (75.0%)	<0.0001
EBV-attributed death	7	2/51 (3.9%)	5/28 (17.8%)	0.0940

N, number of patients.

**Table 8:** Causes of death in analyzed cohort of patients.

Cause of death	Total (N=276)	Group A (N=154)	Group B (N=122)	p-value
EBV-related deaths	7 (2.5%)	6 (3.9%)	1 (0.8%)	0.3226
Other complication	55 (19.9%)	33 (21.4%)	22 (18.0%)	0.8752
Total NRM	62 (23.4%)	39 (25.3%)	23 (18.8%)	0.2445
Progression	30 (10.9%)	22 (14.3%)	8 (6.6%)	0.4658
Total	92 (33.3%)	61 (39.6%)	31 (25.4%)	0.2490

NRM, non-relapse mortality.

### Transplant outcomes

#### Overall survival

Overall survival at 2 years was lower in patients with EBV-PTLD then in patients without EBV-PTLD (OS=0.33 vs OS=0.65; p=0.0010) (**Figure 1F**). A total number of 92 deaths occurred during follow-up, including 16/24 (66.7%) patients with EBV-PTLD (with 7 deaths attributed to PTLD) and 76/252 (30.2%) without EBV-PTLD; 25% of deaths in the group of patients with EBV-PTLD occurred within the first 112 days after HCT, while 25% of deaths in the group of patients without EBV-PTLD occurred within 357 days after HCT. The presence of csEBV-DNA-emia had no impact on transplant outcomes (overall survival, event-free survival, relapse incidence; data not shown), however patients in group B (on prophylactic rituximab), with a lower incidence of csEBV-DNA-emia had a lower incidence of cGVHD (**Table 2**).

#### Relapse

Overall, 55 out of 225 (24.4%) patients with malignant disease relapsed after HCT, including 9/23 (39.1%) with EBV-PTLD and 46/202 (22.8%) without EBV-PTLD (OR=2.1; 95%CI=0.9-6.1; p=0.083). **Figure 1G** shows the incidence of relapse according to the presence of EBV-PTLD (0.72 vs 0.35; p=0.024).

### Risk factor analysis for overall survival after HCT

In univariate analysis, factors contributing to worse overall survival included: presence of EBV-PTLD (HR=2.5; 95%CI=1.52-4.21; p=0.0010), diagnosis of malignant disease (HR=2.1; 95%CI=1.08-4.03; p=0.0303), alternative donor type (HR=1.75; 95%CI=1.04-3.02; p=0.0396), stem cell source (PB; HR=1.86; 95%CI=1.14-3.04; p=0.0106), >CR1 (HR=1.70; 95%CI=1.08-2.66; p=0.0396), HCT number >1 (HR=1.56; 95%CI=1.22-2.19; p=0.0064), IFD after HCT (HR=2.7; 95%CI=1.91-4.07; p<0.0001), and EBV-DNA viral load ≥10^5^ copies/mL (HR=1.5; 95%CI=1.05-2.89; p=0.0484) (**Supplementary Table S1**). Prophylactic use of rituximab showed a trend toward better overall survival both in Kaplan-Meier analysis (p=0.0717) (**Figure 1H**) and the Cox model (HR=0.66; 95%CI=0.43-1.03; p=0.0687).

In a multivariate analysis, adverse prognostic factors for overall survival in patients after allo-HCT were diagnosis of a malignant disease (p=0.0150), >CR1 of the primary disease (p=0.0041), EBV-PTLD (p=0.0025) and IFI after HCT (p<0.0001) (**Table 9**). Factors that did not reach significance included: age category, rituximab prophylaxis, number of HCT, type of donor, and type of conditioning.

**Table 9:** Multivariate analysis of risk factors for overall survival.

Parameter		Number of patients (deaths; %)	OS	HR (95%CI)	p-value
Diagnosis	Malignant	225 (83; 36.9%)	0.57 ± 0.04	2.56 (1.25-5.18)	0.0150
	Non-malignant	51 (9; 17.6%)	0.82 ± 0.06	1.00	
Status of remission	CR>1	157 (36; 22.9%)	0.56 ± 0.04	1.92 (1.22-3.07)	0.0041
	CR1	119 (56; 47.1%)	0.70 ± 0.05	1	
EBV-PTLD	Yes	24 (16; 66.7%)	0.33 ± 0.10	2.13 (1.30-3.35)	0.0025
	No	252 (76; 30.2%)	0.65 ± 0.04	1	
IFI after HCT	Yes	75 (42; 56.0%)	0.36 ± 0.06	2.44 (1.58-3.70)	<0.0001
	No	201 (50; 24.9%)	0.71 ± 0.04	1	

IFI, invasive fungal infection.

### Causes of death

Overall 92/276 (33.3%) deaths occurred; 7 (2.5%) patients due to EBV-related complications, 30 (10.9%) patients died due to the progression of the primary disease, and 55 (19.9%) died due to other complications (**Table 8**).

### Safety of rituximab in prophylactic, preemptive and targeted treatment

Over a period of 13 years, overall 276 reduced prophylactic doses, 340 full doses in pre-emptive and/or targeted treatment of csEBV-DNA-emia/EBV-PTLD and a total of 35 intrathecal rituximab doses were administered. All but one administration was well-tolerated by all patients without symptomatic adverse events. An episode of short seizures was observed after the third intrathecal rituximab infusion in only one patient. The symptoms resolved immediately after diazepam administration. The long-term effects of rituximab on B-cell function were not analyzed in this study.

## Discussion

This study aimed to show the impact of the prophylactic use of rituximab against EBV-related complications in a large pediatric cohort undergoing allo-HCT. We showed that the administration of a single low dose of rituximab on day +5 resulted in significant reduction in the incidence of csEBV-DNA-emia, and significant reduction in the incidence of EBV-PTLD. No decrease in non-relapse mortality was observed, however a trend towards 34% reduction of risk of death in patients after anti-EBV prophylaxis with rituximab was noted in univariate analysis. These results support the concept of the prophylactic use of rituximab in pediatric population. In comparison to study of Dominietto et al. (15) in adults, we have shown not only decrease of incidence of csEBV-DNA-emia, but also the incidence of EBV-PTLD. In two other studies in adults on the prophylactic role of rituximab, Van Besien et al. (16) and Patel et al. (17) used one dose of rituximab in pretransplant period. This prophylaxis significantly decreased the percentage of patients with detectable EBV-DNA-emia and EBV-PTLD incidence. Nevertheless, the impact on mortality due to EBV-PTLD was non-significant, although there was a trend in favor of prophylaxis. Similarly, although prophylaxis with rituximab did not change overall survival, a non-significant trend towards 50% reduction of mortality was observed. This trend can results from significant decrease of PTLD incidence in patients on prophylaxis. Our findings are specific for pediatric population only because of the specificity of having high rate of EBV infection in the first year of life and a shorter latency in comparison to adults. Genomic and immunologic profiling of PTLD can provide additional more insights into the nature of this disease (25, 26).

A disadvantage of prophylaxis with rituximab is the exposure of its toxicity in all transplant recipients, however the use of a reduced dose can be advantageous. Although administration of rituximab is safe and no immediate major adverse events were reported, the risk of prolonged hypogammaglobulinemia or neutropenia is a concern in terms of increased risk of infections (27, 28). In our cohort we did not observe these complications in patients treated prophylactically with rituximab, as well as CMV or fungal infection, however rate of patients with CD19 recovery on day +180 was lower in this cohort. Storek and Lindsay (27) recommend that prophylaxis with one dose of rituximab in the peri-transplant period could be a reasonable strategy for adult patients with a high risk of development and mortality due to PTLD. In children further study on long-term complications are necessary.

Another new finding of this study is an increased relapse incidence in patients who were treated for EBV-PTLD. This observation is completely new and highly unexpected. Possibly, it can reflect patient susceptibility to cancerogenesis. Nowadays although some studies have indicated prolonged or long-term toxicity of rituximab (29, 30), it should be underlined that rituximab is the gold standard in treatment of EBV-PTLD in HCT setting (3, 7, 27, 31–33).

Current ECIL guidelines recommend monitoring for EBV viremia in patients at high risk of developing EBV-PTLD, and initiating pre-emptive treatment when EBV-DNA-emia is diagnosed (7). We have shown that prophylaxis with rituximab in children after allo-HCT was effective in terms of decreasing the incidence of csEBV-DNA-emia and EBV-PTLD. Majority of our patients were after MUD transplant and were administered ATG, thus according to ECIL guidelines could be classified as high risk group (7).

We have shown that the risk of developing of PTLD increases with higher EBV viremia. There are limited pediatric data on the risk factors for the progression of EBV-DNA-emia to EBV-PTLD. Our study showed that EBV reactivation before day +100 after HCT increases the risk of developing EBV-PTLD. It was also found that an increase or persistence of the viral load after 2 weeks of treatment with rituximab and the indication for a subsequent dose are strong predictors of disease development. In addition, the analysis showed that the risk of developing EBV-PTLD increased with increasing EBV-DNA viremia. A single-center study of a group of 59 patients showed 90% sensitivity and specificity of the threshold value of 10^5^ copies/mL for the development of EBV-PTLD (34). Taking together, these analyses showed that the EBV-DNA viral load was an important predictor of the development of EBV-PTLD, and early monitoring and prompt treatment can prevent uncontrolled viral replication and the development of EBV disease.

Analysis of risk factors has shown that the occurrence of EBV infection depends on many complex interactions between the underlying disease and the transplantation procedure, source of hematopoietic cells, donor type and many others. The risk of developing EBV-PTLD is proportional to the degree of depletion and impairment of T-lymphocyte function (35). It is very difficult to create a uniform model for assessing the risk of EBV-PTLD, and the scoring systems used in the literature to assess the risk of developing EBV-PTLD are not commonly used in clinical practice. On the other hand, some reports suggest protective role of mycofenolate mofetil (36) or sirolimus (37) or post-transplant high-dose cyclophosphamide (38) in prevention of EBV infection.

The management of EBV infections in patients after HCT consists of three main strategies: prophylaxis, preemptive treatment and treatment. Therapeutic methods used in the prevention and treatment of EBV-PTLD include: rituximab administration, reduction of immunosuppression, cell therapy with EBV-specific cytotoxic T cells, donor lymphocyte infusion and chemotherapy.

Rituximab when used in preemptive therapy is effective in more than 90% of patients, and when administered in targeted EBV-PTLD therapy, it is effective in nearly 65% of patients (4, 7). In our study, pre-emptive treatment with rituximab was used in a total of 76% (60/79) of patients, showing an efficacy rate of 88.4% (relative to the number of deaths due to EBV-PTLD). The use of preemptive therapy at a viral load of ≥10^4^ copies/mL was associated with reaching EBV-DNA-emia negativity in 92.3% of patients, while starting at viral load ≥10^5^ copies/mL resulted in EBV-DNA-emia negativity in 82.1%. The development of EBV-PTLD was significantly more frequent with the initiation of pre-emptive treatment at a viral load ≥10^5^ copies/mL than at a viral load ≥10^4^ copies/mL. Nevertheless, the number of deaths and OS were comparable between the two groups. This finding suggested that even if the effectiveness of pre-emptive treatment is higher at a viral load ≥10^4^ copies/mL, a significant proportion of patients die from other causes. The efficacy of targeted EBV-PTLD therapy was 70.8%.

In our analysis, 92 (33%) deaths were recorded: 7 deaths due to EBV infection, 30 due to progression of the underlying disease and 55 due to other complications. Mortality in the group of patients with EBV-PTLD was 29%, which is a substantial achievement compared to the 86% mortality rate reported in the literature over 20 years ago (8). In the largest multicenter study to date (9), a mortality rate of 31% due to EBV-PTLD was comparable to that in our study. The highest number of deaths was recorded within the first year (1-year OS=0.72). Compared to the previously mentioned multicenter study (9) in which the 3-year OS was 47.3%, in our pediatric study the overall survival rate was higher reaching 62%. There was no impact of csEBV-DNA-emia on overall survival, but OS was significantly lower in patients with EBV-PTLD than in patients without EBV-PTLD. As in the majority of studies published over the last decade, 20-30% of EBV-PTLD patients are not cured with rituximab in the HCT setting, it strongly underlines the emerging need for the use of newer therapies in refractory PTLD (14). EBV-CTLs are at the highest hope for standard use (39–43), although other therapies have been reported in clinical practice both in hematopoietic and solid organ transplant settings (44–48).

Our study has several limitations. This is a retrospective analysis, with some differences in the distribution of patients according to the strategy regimen; nevertheless more high risk factors were detected in the group of patients who received prophylaxis with rituximab, so the effect of prophylaxis was even more pronounced. The monitoring for EBV-DNA-emia in Group A was done in various intervals, so we cannot exclude very low risk of possible underestimation of EBV viremia in group A, i.e. patients without rituximab prophylaxis, due to possible transient self-limiting low-grade EBV viremia. Nevertheless, even with the limitation of lower number of PCR tests per patient in group A, the prevalence of csEBV-DNA-emia was significantly higher in this group. Another limitation is low rate of proven diagnosis of PTLD. This is a common issue of diagnostic process of PTLD in pediatric HCT setting due to following factors: usually quick progression of the disease and necessity of quick therapeutic intervention, logistic issues related to invasive procedure performed in general anesthesia, and lack of agreement of parents who are conscious about using criterium of probable diagnosis. This observation is confirmed in recent EBMT survey (48), as the low rate of biopsy-confirmed EBV-PTLD was reported in pediatric setting, when compared to adult patients.

We did not analyze the risk of infection after prophylactic use of rituximab, which is a topic outside the scope of this study. However, data from existing studies suggest that with one prophylactic dose of rituximab, the risk of infection is not increased (15–17). We did not see an impact of use of alemtuzumab on EBV-related complications, but this could be the result of a small group of patients treated with this compound. Also, pre-transplant EBV serology of recipient and donor did not show the impact on transplant outcomes or development of EBV viremia and PTLD. There is a concern that after treatment with rituximab, the downregulation of CD20 can occur, but we did not observe CD20 negativity on diagnosis of PTLD in our patients who received prophylactic rituximab.

In conclusion, the introduction of single-dose rituximab administration during the peri-transplant period significantly reduced the number of EBV infections. Prevention, monitoring of EBV-DNA-emia and early treatment are the most important elements of the management of EBV infections, which have resulted in a significant decrease in mortality from PTLD over last two decades. Results of this study indicate option of prophylactic use of rituximab.

## Data availability statement

The raw data supporting the conclusions of this article will be made available by the authors, without undue reservation.

## Ethics statement

The studies involving humans were approved by Komisja Bioetyczna, Collegium Medicum w Bydgoszczy (Bioethical Committee). Decisions KB 499/2014 and KB 696/2017. The studies were conducted in accordance with the local legislation and institutional requirements. Written informed consent for participation in this study was provided by the participants’ legal guardians/next of kin.

## Author contributions

AM: Data curation, Formal analysis, Investigation, Methodology, Validation, Visualization, Writing – original draft, Writing – review & editing. MP: Data curation, Formal analysis, Methodology, Validation, Writing – original draft, Writing – review & editing. MD: Data curation, Formal analysis, Methodology, Validation, Writing – original draft, Writing – review & editing. KC: Data curation, Formal analysis, Methodology, Validation, Writing – original draft, Writing – review & editing. MR: Data curation, Formal analysis, Methodology, Validation, Writing – original draft, Writing – review & editing. RD: Data curation, Formal analysis, Methodology, Validation, Writing – original draft, Writing – review & editing. TB: Formal analysis, Investigation, Methodology, Writing – original draft, Writing – review & editing. JS: Conceptualization, Data curation, Formal analysis, Funding acquisition, Investigation, Methodology, Project administration, Resources, Software, Supervision, Validation, Visualization, Writing – original draft, Writing – review & editing.

## References

[1] Cohen JI. Epstein-Barr virus infection. N Engl J Med. (2000) 343:481–92. doi: 10.1056/NEJM200008173430707 10944566

[2] Styczynski J Tridello G Gil L Ljungman P Hoek J Iacobelli S. Impact of donor Epstein-Barr Virus serostatus on the incidence of graft-versus-host disease in patients with acute leukemia after hematopoietic stem-cell transplantation: a study from the Acute Leukemia and Infectious Diseases Working Parties of the European Society for Blood and Marrow Transplantation. J Clin Oncol. (2016) 34:2212–20. doi: 10.1200/JCO.2015.64.2405 27091716

[3] Kania SP Silva JMF Charles OJ Booth J Cheung SYA Yates JWT. Epstein-Barr Virus reactivation after paediatric haematopoietic stem cell transplantation: risk factors and sensitivity analysis of mathematical model. Front Immunol. (2022) 13:903063. doi: 10.3389/fimmu.2022.903063 35903096 PMC9314642

[4] Styczynski J. Managing post-transplant lymphoproliferative disorder. Expert Opin Orphan Drugs. (2017) 5:19–35. doi: 10.1080/21678707.2017.1262256

[5] Silva JM Alves CEC Pontes GS. Epstein-Barr virus: the mastermind of immune chaos. Front Immunol. (2024) 15:1297994. doi: 10.3389/fimmu.2024.1297994 38384471 PMC10879370

[6] Kanakry JA Hegde AM Durand CM Massie AB Greer AE Ambinder RF. The clinical significance of EBV DNA in the plasma and peripheral blood mononuclear cells of patients with or without EBV diseases. Blood. (2016) 127:2007–17. doi: 10.1182/blood-2015-09-672030 PMC4841041 26744460

[7] Styczynski J van der Velden W Fox CP Engelhard D de la Camara R Cordonnier C. Management of Epstein-Barr Virus infections and post-transplant lymphoproliferative disorders in patients after allogeneic hematopoietic stem cell transplantation: Sixth European Conference on Infections in Leukemia (ECIL-6) guidelines. Haematologica. (2016) 101:803–11. doi: 10.3324/haematol.2016.144428 PMC5004459 27365460

[8] Curtis RE Travis LB Rowlings PA Socie G Kingma DW Banks PM. Risk of lymphoproliferative disorders after bone marrow transplantation: a multi-institutional study. Blood. (1999) 94:2208–16. 10498590

[9] Styczynski J Gil L Tridello G Ljungman P Donnelly JP van der Velden W. Response to rituximab-based therapy and risk factor analysis in Epstein Barr Virus-related lymphoproliferative disorder after hematopoietic stem cell transplant in children and adults: a study from the Infectious Diseases Working Party of the European Group for Blood and Marrow Transplantation. Clin Infect Dis. (2013) 57:794–802. doi: 10.1093/cid/cit391 23771985

[10] Fox CP Burns D Parker AN Peggs KS Harvey CM Natarajan S. EBV-associated post-transplant lymphoproliferative disorder following *in vivo* T-cell-depleted allogeneic transplantation: clinical features, viral load correlates and prognostic factors in the rituximab era. Bone Marrow Transplant. (2014) 49:280–6. doi: 10.1038/bmt.2013.170 24212561

[11] Kinzel M Dowhan M Kalra A Williamson TS Dabas R Jamani K. Risk Factors for the incidence of and the mortality due to post-transplant lymphoproliferative disorder after hematopoietic cell transplantation. Transplant Cell Ther. (2022) 28:53.e1–53.e10. doi: 10.1016/j.jtct.2021.09.021 34607072

[12] Ullah A Lee KT Malham K Yasinzai AQK Khan I Asif B. Post-transplant lymphoproliferative disorder (PTLD) in the US population: demographics, treatment characteristics, and survival analysis. Cureus. (2023) 15:e39777. doi: 10.7759/cureus.39777 37398803 PMC10312545

[13] Papalexandri A Gavriilaki E Vardi A Kotsiou N Demosthenous C Constantinou N. Pre-emptive use of rituximab in Epstein-Barr Virus reactivation: incidence, predictive factors, monitoring, and outcomes. Int J Mol Sci. (2023) 24:16029. doi: 10.3390/ijms242216029 38003218 PMC10671524

[14] Socié G Barba P Barlev A Sanz J García-Cadenas I Chevallier P. Outcomes for patients with EBV-positive PTLD post-allogeneic HCT after failure of rituximab-containing therapy. Bone Marrow Transplant. (2024) 59:52–8. doi: 10.1038/s41409-023-02127-9 PMC10781634 37865719

[15] Dominietto A Tedone E Soracco M Bruno B Raiola AM Van Lint MT. *In vivo* B-cell depletion with rituximab for alternative donor hemopoietic SCT. Bone Marrow Transplant. (2012) 47:101–6. doi: 10.1038/bmt.2011.28 21460867

[16] Van Besien K Bachier-Rodriguez L Satlin M Brown MA Gergis U Guarneri D. Prophylactic rituximab prevents EBV PTLD in haplo-cord transplant recipients at high risk. Leuk Lymphoma. (2019) 60:1693–6. doi: 10.1080/10428194.2018.1543877 30741059

[17] Patel C Pasciolla M Abramova R Salerno D Gomez-Arteaga A Shore TB. Pre-hematopoietic stem cell transplantation rituximab for Epstein-Barr Virus and post-lymphoproliferative disorder prophylaxis in alemtuzumab recipients. Transplant Cell Ther. (2023) 29:132 e1–5. doi: 10.1016/j.jtct.2022.10.023 36334653

[18] Styczynski J Reusser P Einsele H de la Camara R Cordonnier C Ward KN. Management of HSV, VZV and EBV infections in patients with hematological Malignancies and after SCT: guidelines from the Second European Conference on Infections in Leukemia. Bone Marrow Transplant. (2009) 43:757–70. doi: 10.1038/bmt.2008.386 19043458

[19] Swerdlow SH Campo E Pileri SA Harris NL Stein H Siebert R. The 2016 revision of the World Health Organization classification of lymphoid neoplasms. Blood. (2016) 127:2375–90. doi: 10.1182/blood-2016-01-643569 PMC4874220 26980727

[20] Campo E Jaffe ES Cook JR Quintanilla-Martinez L Swerdlow SH Anderson KC. The international consensus classification of mature lymphoid neoplasms: a report from the Clinical Advisory Committee. Blood. (2022) 140:1229–53. doi: 10.1182/blood.2022015851 PMC9479027 35653592

[21] Alaggio R Amador C Anagnostopoulos I Attygalle AD Araujo IBO Berti E. The 5th edition of the world health organization classification of haematolymphoid tumours: lymphoid neoplasms. Leukemia. (2022) 36:1720–48. doi: 10.1038/s41375-022-01620-2 PMC9214472 35732829

[22] Styczyński J Czyżewski K Ussowicz M Helbig G Sobczyk-Kruszelnicka M Łojko A. Antimicrobial prophylaxis in patients after hematopoietic cell transplantation: results of a survey of the Polish Federation of Bone Marrow Transplant Centers. Acta Haematologica Pol. (2020) 51:183–6. doi: 10.2478/ahp-2020-0032

[23] De Pauw B Walsh TJ Donnelly JP Stevens DA Edwards JE Calandra T. Revised definitions of invasive fungal disease from the European Organization for Research and Treatment of Cancer/Invasive Fungal Infections Cooperative Group and the National Institute of Allergy and Infectious Diseases Mycoses Study Group (EORTC/MSG) Consensus Group. Clin Infect Dis. (2008) 46:1813–21. doi: 10.1086/588660 PMC2671227 18462102

[24] Donnelly JP Chen SC Kauffman CA Steinbach WJ Baddley JW Verweij PE. Revision and update of the consensus definitions of invasive fungal disease from the European Organization for Research and Treatment of Cancer and the Mycoses Study Group Education and Research Consortium. Clin Infect Dis. (2020) 71:1367–76. doi: 10.1093/cid/ciz1008 PMC7486838 31802125

[25] Veltmaat N Zhong Y de Jesus FM Tan GW Bult JAA Terpstra MM. Genomic profiling of post-transplant lymphoproliferative disorders using cell-free DNA. J Hematol Oncol. (2023) 16:104. doi: 10.1186/s13045-023-01500-x 37705050 PMC10500745

[26] Compagno F Basso S Panigari A Bagnarino J Stoppini L Maiello A. Management of PTLD after hematopoietic stem cell transplantation: immunological perspectives. Front Immunol. (2020) 11:567020. doi: 10.3389/fimmu.2020.567020 33042147 PMC7526064

[27] Storek J Lindsay J. Rituximab for posttransplant lymphoproliferative disorder - therapeutic, preemptive, or prophylactic? Bone Marrow Transplant. (2024) 59:6–11. doi: 10.1038/s41409-023-02155-5 38001229

[28] Griffin JM Healy FM Dahal LN Floisand Y Woolley JF. Worked to the bone: antibody-based conditioning as the future of transplant biology. J Hematol Oncol. (2022) 15:65. doi: 10.1186/s13045-022-01284-6 35590415 PMC9118867

[29] Kinzel M Kalra A Khanolkar RA Williamson TS Li N Khan F. Rituximab toxicity after preemptive or therapeutic administration for post-transplant lymphoproliferative disorder. Transplant Cell Ther. (2023) 29:43 e1–8. doi: 10.1016/j.jtct.2022.10.013 36273783

[30] McGlynn MC Brady K Healey JM Dharnidharka VR Ybarra AM Stoll J. Late effects in survivors of post-transplant lymphoproliferative disease. Pediatr Blood Cancer. (2024) 71:e30777. doi: 10.1002/pbc.30777 37988230

[31] Amengual JE Pro B. How I treat posttransplant lymphoproliferative disorder. Blood. (2023) 142:1426–37. doi: 10.1182/blood.2023020075 PMC10731918 37540819

[32] Enok Bonong PR Zahreddine M Buteau C Duval M Laporte L Lacroix J. Factors associated with post-transplant active epstein-barr virus infection and lymphoproliferative disease in hematopoietic stem cell transplant recipients: a systematic review and meta-analysis. Vaccines (Basel). (2021) 9. doi: 10.3390/vaccines9030288 PMC8003684 33808928

[33] Marjanska A Styczynski J. Who is the patient at risk for EBV reactivation and disease: expert opinion focused on post-transplant lymphoproliferative disorders following hematopoietic stem cell transplantation. Expert Opin Biol Ther. (2023) 23:539–52. doi: 10.1080/14712598.2023.2196366 36971380

[34] Laberko A Bogoyavlenskaya A Shelikhova L Shekhovtsova Z Balashov D Voronin K. Risk factors for and the clinical impact of cytomegalovirus and epstein-barr virus infections in pediatric recipients of TCR-alpha/beta- and CD19-depleted grafts. Biol Blood Marrow Transplant. (2017) 23:483–90. doi: 10.1016/j.bbmt.2016.12.635 28039080

[35] Van der Velden WJ Mori T Stevens WB de Haan AF Stelma FF Blijlevens NM. Reduced PTLD-related mortality in patients experiencing EBV infection following allo-SCT after the introduction of a protocol incorporating pre-emptive rituximab. Bone Marrow Transplant. (2013) 48:1465–71. doi: 10.1038/bmt.2013.84 23749107

[36] Enok Bonong PR Buteau C Duval M Lacroix J Laporte L Tucci M. Risk factors for post-transplant Epstein-Barr virus events in pediatric recipients of hematopoietic stem cell transplants. Pediatr Transplant. (2021) 25:e14052. doi: 10.1111/petr.14052 34076939

[37] Garcia-Cadenas I Castillo N Martino R Barba P Esquirol A Novelli S. Impact of Epstein Barr virus-related complications after high-risk allo-SCT in the era of pre-emptive rituximab. Bone Marrow Transplant. (2015) 50:579–84. doi: 10.1038/bmt.2014.298 25581404

[38] Kanakry JA Kasamon YL Bolanos-Meade J Borrello IM Brodsky RA Fuchs EJ. Absence of post-transplantation lymphoproliferative disorder after allogeneic blood or marrow transplantation using post-transplantation cyclophosphamide as graft-versus-host disease prophylaxis. Biol Blood Marrow Transplant. (2013) 19:1514–7. doi: 10.1016/j.bbmt.2013.07.013 PMC4051232 23871780

[39] Grzesk E Koltan S Dabrowska A Urbanczyk A Maldyk J Malkowski B. Case report: Cellular therapy for hydroa vacciniforme-like lymphoproliferative disorder in pediatric common variable immunodeficiency with chronic active Epstein-Barr virus infection. Front Immunol. (2022) 13:915986. doi: 10.3389/fimmu.2022.915986 35990691 PMC9390486

[40] Keam SJ. Tabelecleucel: first approval. Mol Diagn Ther. (2023) 27:425–31. doi: 10.1007/s40291-023-00648-z 37016096

[41] Kaeuferle T Krauss R Blaeschke F Willier S Feuchtinger T. Strategies of adoptive T -cell transfer to treat refractory viral infections post allogeneic stem cell transplantation. J Hematol Oncol. (2019) 12:13. doi: 10.1186/s13045-019-0701-1 30728058 PMC6364410

[42] Romero D. Tabelecleucel is effective in EBV-positive lymphoproliferative disease. Nat Rev Clin Oncol. (2024) 21:251. doi: 10.1038/s41571-024-00873-3 38374435

[43] Mahadeo KM Baiocchi R Beitinjaneh A Chaganti S Choquet S Dierickx D. Tabelecleucel for allogeneic haematopoietic stem-cell or solid organ transplant recipients with Epstein-Barr virus-positive post-transplant lymphoproliferative disease after failure of rituximab or rituximab and chemotherapy (ALLELE): a phase 3, multicentre, open-label trial. Lancet Oncol. (2024) 25:376–87. doi: 10.1016/S1470-2045(23)00649-6 38309282

[44] Preiksaitis J Allen U Bollard CM Dharnidharka VR Dulek DE Green M. The IPTA Nashville Consensus Conference on Post-Transplant lymphoproliferative disorders after solid organ transplantation in children: III - Consensus guidelines for Epstein-Barr virus load and other biomarker monitoring. Pediatr Transplant. (2024) 28:e14471. doi: 10.1111/petr.14471 37294621

[45] Green M Squires JE Chinnock RE Comoli P Danziger-Isakov L Dulek DE. The IPTA Nashville consensus conference on Post-Transplant lymphoproliferative disorders after solid organ transplantation in children: II-consensus guidelines for prevention. Pediatr Transplant. (2024) 28:e14350. doi: 10.1111/petr.14350 36369745

[46] Wilkinson JD Allen U Green M Dipchand AI Dharnidharka VR Esquivel CO. The IPTA Nashville consensus conference on post-transplant lymphoproliferative disorders after solid organ transplantation in children: I-Methodology for the development of consensus practice guidelines. Pediatr Transplant. (2024) 28:e14333. doi: 10.1111/petr.14333 36369733

[47] Allen UD Preiksaitis JK AST Infectious Diseases Community of Practice. Post-transplant lymphoproliferative disorders, Epstein-Barr virus infection, and disease in solid organ transplantation: Guidelines from the American Society of Transplantation Infectious Diseases Community of Practice. Clin Transplant. (2019) 33:e13652. doi: 10.1111/ctr.13652 31230381

[48] Styczynski J Tridello G Wendel L Knelange N Cesaro S Gil L. Prevalence, management, and new treatment modalities of EBV-DNA-emia and EBV-PTLD after allo-HCT: survey of Infectious Diseases Working Party EBMT. Bone Marrow Transplant. (2024) 59:59–65. doi: 10.1038/s41409-023-02129-7 37872300

