# Supplementary material for: Impact of prophylaxis with rituximab on EBV-related complications after allogeneic hematopoietic cell transplantation in children
Source: Front Immunol. 2024 Jul 11;15:1427637. doi: 10.3389/fimmu.2024.1427637 (PMC11269116; doi:10.3389/fimmu.2024.1427637)
Supplement: Supplementary file 1 [file Table_1.doc]

**Supplementary Table S1.** Univariate analyses for csEBVi, EBV-PTLD and overall survival.

| Parameter | | Pts (n=276) | csEBV-DNA-emia (n=79) | | | EBV-PTLD (n=24) | | | Overall survival (n=92 deaths) | | |
| --- | --- | --- | --- | --- | --- | --- | --- | --- | --- | --- | --- |
|  |  |  | HR | 95% CI | P | HR | 95% CI | P | HR | 95% CI | P |
| Prophylaxis with rituximab | Yes | 122 | 0.48 | 0.28–0.83 | 0.0078 | 0.23 | 0.08–0.69 | 0.0045 | 1.00 | 0.97–2.32 | 0.0687 |
|  | No | 154 | 1.00 |  |  | 1.00 |  |  | 1.50 |  |  |
| Sex | Female | 105 | 0.79 | 0.46–1.37 | 0.4021 | 1.18 | 0.50–2.77 | 0.7020 | 1.00 | 0.50--1.48 | 0.3213 |
|  | Male | 171 | 1.00 |  |  | 1.00 |  |  | 0.89 |  |  |
| Age | ≥10 | 137 | 1.22 | 0.72–2.06 | 0.4580 | 1.47 | 0.63–3.44 | 0.3726 | 1.00 | 0.43–0.97 | 0.0236 |
|  | <10 | 139 | 1.00 |  |  | 1.00 |  |  | 0.64 |  |  |
| Diagnosis of acute lymphoblastic leukemia (ALL) | Yes | 117 | 1.00 | 0.49–1.42 | 0.5024 | 1.41 | 0.61–3.28 | 0.4299 | 1.00 | 0.65–1.49 | 0.4679 |
|  | No | 159 | 0.83 |  |  | 1.00 |  |  | 0.98 |  |  |
| Diagnosis | Malignant | 225 | 1.14 | 0.58–2.25 | 0.6924 | 5.87 | 0.78–44.12 | 0.0842 | 1.00 | 0.24–0.94 | 0.0303 |
|  | Non-malignant | 51 | 1.00 |  |  | 1.00 |  |  | 0.47 |  |  |
| Status of remission | CR1* | 157 | 0.99 | 0.59–1.69 | 0.9868 | 0.61 | 0.26–1.42 | 0.3532 | 1.00 | 1.08–2.66 | 0.0396 |
|  | >CR1 | 119 | 1.00 |  |  | 1.00 |  |  | 1.70 |  |  |
| HCT number | 1 | 238 | 1.00 | 0.76–3.20 | 0.2274 | 1.00 | 0.84–6.23 | 0.0923 | 1.00 | 0.43–0.87 | 0.0064 |
|  | >1 | 38 | 1.55 |  |  | 2.29 |  |  | 0.64 |  |  |
| Type of donor 1 | MUD | 195 | 1.00 | – | – | 1.00 | – | ­0.0463 |  |  | NS |
|  | MMUD | 9 | 1.49 | 0.39–5.75 | 0.5592 | 2.37 | 0.46–2.15 |  |  |  |  |
|  | MFD | 67 | 0.22 | 0.09–0.50 | 0.0005 | 0.13 | 0.02–0.95 |  |  |  |  |
|  | HAPLO | 5 | 0.00 | – | 0.9963 | 0.00 | – |  |  |  |  |
| Type of donor 2 | MFD | 67 | 0.21 | 0.09–0.49 | 0.0010 | 0.12 | 0.02–0.90 | 0.0144 | 0.57 | 0.33–0.97 | 0.0396 |
|  | MUD/MMUD | 204 | 1.00 |  |  | 1.00 |  |  | 1.00 |  |  |
| Cell source 1 | PB | 178 | 1.00 | – | – | 1.00 | – | 0.0143 |  |  | NS |
|  | BM | 95 | 0.49 | 0.27–0.89 | 0.0174 | 0.15 | 0.04–0.66 |  |  |  |  |
|  | CB | 3 | 0.00 | – | 0.9999 | 0.00 | – |  |  |  |  |
| Cell source 2 | PB | 178 | 2.03 | 1.12–3.68 | 0.0174 | 6.56 | 1.51–28.53 | 0.0144 | 1.86 | 1.14–3.04 | 0.0106 |
|  | BM | 95 | 1.00 |  |  | 1.00 |  |  | 1.00 |  |  |
| EBV serostatus | R+/D+ | 181 | 1.00 | – | 0.1788 | 1.00 | – | 0.5347 | 1.00 | – | 0.9474 |
|  | R+/D– | 20 | 0.48 | 0.15–1.49 |  | 1.01 | 0.22–4.69 |  | 1.26 | 0.15-10.7 |  |
|  | R–/D+ | 14 | 1.92 | 0.64–5.72 |  | 2.47 | 0.63–9.88 |  | 1.01 | 0.12–8.3 |  |
|  | R–/D– | 3 | 0.00 | – |  | 0.00 | – |  | 1.14 | 0.16–8.2 |  |
| Recipient EBV IgG | R+ | 208 | 0.77 | 0.31–1.97 | 0.5840 | 1.00 | 0.27–5.56 | 0.3542 |  |  | NS |
|  | R– | 21 | 1.00 |  |  | 1.22 |  |  |  |  |  |
| Donor EBV IgG | D+ | 212 | 2.39 | 0.78–7.35 | 0.1157 | 1.00 | 0.17–2.39 | 0.5748 |  |  | NS |
|  | D– | 23 | 1.00 |  |  | 0.64 |  |  |  |  |  |
| CMV serostatus | R+/D+ | 117 | 1.00 | – | 0.1367 | 1.00 | – | 0.9655 | 1.00 | – | 0.1915 |
|  | R+/D– | 101 | 0.89 | 0.49–1.61 |  | 1.05 | 0.41–2.69 |  | 2.27 | 0.96–5.35 |  |
|  | R–/D+ | 18 | 2.44 | 0.89–6.68 |  | 0.63 | 0.08–5.24 |  | 1.68 | 0.51–5.50 |  |
|  | R–/D– | 31 | 0.59 | 0.22–1.56 |  | 1.15 | 0.30–4.45 |  | 2.28 | 0.96–5.40 |  |
| Recipient CMV IgG | R+ | 220 | 0.89 | 0.45–1.75 | 0.7336 | 1.00 | 0.73–3.98 | 0.2074 |  |  | NS |
|  | R– | 49 | 1.00 |  |  | 1.22 |  |  |  |  |  |
| Donor CMV IgG | D+ | 139 | 1.47 | 0.86–2.50 | 0.1566 | 1.00 | 0.42–2.22 | 0.9251 |  |  | NS |
|  | D– | 134 | 1.00 |  |  | 0.96 |  |  |  |  |  |
| ABO Blood group compatibility | Yes | 116 | 0.79 | 0.46–1.35 | 0.3875 | 1.00 | 0.73–3.98 | 0.2074 | 1.00 | 0.77–1.77 | 0.3901 |
|  | No | 160 | 1.00 |  |  | 1.71 |  |  | 1.16 |  |  |
| Rh blood group compatibility | Yes | 210 | 0.99 | 0.52–1.88 | 0.9729 | 1.00 | 0.29–1.89 | 0.5277 | 1.00 | 0.40–1.15 | 0.6556 |
|  | No | 66 | 1.00 |  |  | 0.74 |  |  | 0.68 |  |  |
| Conditioning 1 | MAC | 163 | 1.00 | 0.52–1.51 | 0.6539 | 1.00 | 0.42–2.25 | 0.9398 | 1.00 | 1.06–2.41 | 0.0259 |
|  | RIC | 113 | 0.89 |  |  | 0.97 |  |  | 1.60 |  |  |
| Conditioning 2 | TBI | 46 | 1.00 | 0.72–2.78 | 0.3113 | 1.00 | 0.66–4.75 | 0.2516 | 1.00 | 0.96-3.38 | 0.0674 |
|  | Chemotherapy | 230 | 1.41 |  |  | 1.77 |  |  | 1.80 |  |  |
| T-depletion *in vivo* | Yes | 223 | 13.13 | 3.14–54.90 | <0.0001 | 1.00 | undefined | 0.0046 | 1.00 | 0.49–1.40 | 0.4825 |
|  | No | 53 | 1.00 |  |  | 0.00 |  |  | 0.83 |  |  |
| Type of T‑depletion | ATG | 212 | 1.00 | 0.48–5.49 | 0.4344 | 1.00 | undefined | 0.2375 |  |  | NS |
|  | Alemtuzumab | 10 | 1.62 |  |  | 0.00 |  |  |  |  |  |
| Use of MMF in prophylaxis of GVHD | Yes | 28 | 1.20 | 0.52-2.79 | 0.663 | 0.00 | undefined | 0.148 | 0.94 | 0.40-2.17 | 0.887 |
|  | No | 248 | 1.00 |  |  | 1.00 |  |  | 1.00 |  |  |
| CMV infection | Yes | 110 | 1.50 | 0.88–2.54 | 0.1363 | 0.74 | 0.30–1.79 | 0.4947 | 1.00 | 0.56–1.29 | 0.2187 |
|  | No | 166 | 1.00 |  |  | 1.00 |  |  | 0.85 |  |  |
| BKV infection | Yes | 45 | 1.66 | 0.85–3.24 | 0.1375 | 0.71 | 0.20–2.52 | 0.4268 | 1.00 | 0.53-­1.52 | 0.2420 |
|  | No | 231 | 1.00 |  |  | 1.00 |  |  | 0.90 |  |  |
| IFI before HCT | Yes | 22 | 0.93 | 0.35–2.48 | 0.8839 | 0.48 | 0.06–3.75 | 0.4082 | 1.00 | 0.43­-1.68 | 0.8408 |
|  | No | 254 | 1.00 |  |  | 1.00 |  |  | 0.85 |  |  |
| IFI after HCT | Yes | 75 | 1.48 | 0.84–2.63 | 0.1748 | 1.69 | 0.70–4.06 | 0.2340 | 1.00 | 0.24­-0.54 | <0.0001 |
|  | No | 201 | 1.00 |  |  | 1.00 |  |  | 0.36 |  |  |
| aGvHD | Yes | 96 | 1.35 | 0.77–2.35 | 0.2897 | 0.73 | 0.28–1.92 | 0.5197 | 1.00 | 0.55­-1.30 | 0.3281 |
|  | No | 180 | 1.00 |  |  | 1.00 |  |  | 0.84 |  |  |
| cGvHD (176 pts evaluable) | Yes | 40 | 2.03 | 1.03–3.99 | 0.0366 | 1.09 | 0.35–3.38 | 0.5330 | 1.00 | 0.63-­1.90 | 0.1708 |
|  | No | 136 | 1.00 |  |  | 1.00 |  |  | 1.10 |  |  |
| Time from HCT to EBV infection | <100 days | 53 | - | - | NA | 48.12 | 13.58-170.56 | 0.0088 | - | - | NA |
|  | ≥100 days | 26 | - | - |  | 1.00 |  |  | - | - |  |
| Max value of EBV-DNA-emia (c/mL) | <10^5^ | 248 | - | - | NA | 0.05 | 0.02-­0.17 | <0.0001 | 1.00 | 0.37­-0.96 | 0.0468 |
|  | ≥10^5^ | 28 | - | - |  | 1.00 |  |  | 0.66 |  |  |
| Good response after 2 doses of rituximab in preemptive therapy | Yes | 52 | - | - | NA | 0.00 | undefined | <0.0001 | - | - | NA |
|  | No | 27 | - | - |  | 1.00 |  |  | - | - |  |
| EBV-DNA-emia | Yes | 79 | - | - | NA | 4.4 | 1.5-13.2 | 0.0045 | 1.00 | 0.57–1.37 | 0.5861 |
|  | No | 197 | - | - |  | 1.0 |  |  | 0.89 |  |  |
| EBV-PTLD | Yes | 24 | - | - | NA | - | - | NA | 1.00 | 0.23–0.68 | 0.0010 |
|  | No | 252 | - | - |  | - | - |  | 0.40 |  |  |

** non-malignant diseases were classified as CR1; HR, hazard risk; 95%CI, 95% confidence interval; ND, no data; NS, not significant; SAA, severe aplastic anemia; BMF, bone marrow failure; IEI, inborn errors of immunity; CR, complete remission; MFD, matched family donor; MUD, matched unrelated donor; MMUD, mismatched unrelated donor; HAPLO, haploidentical donor; MAC, myeloablative conditioning; RIC, reduced intensity of conditioning; TBI, total body irradiation; ATG, anti-thymocyte globulin; CsA, cyclosporin A; PTCy, post-transplant cyclophosphamide; GvHD, graft-versus-host disease; aGvHD, acute GvHD; cGvHD, chronic GvHD; R, recipient; D, donor; BKV, polyoma virus BKV; CMV, cytomegalovirus; MMF, mycofenolate mofetil*
